# Microsurgical Management of Spinal Extradural Arachnoid Cyst: Case Report and Review of Literature

**DOI:** 10.7759/cureus.95148

**Published:** 2025-10-22

**Authors:** Vikrant Keshri, Sarav Bamania

**Affiliations:** 1 Neurosurgery, SBKS Medical Institute and Research Centre, Sumandeep Vidyapeeth Deemed University, Vadodara, IND

**Keywords:** laminectomy, mri imaging, neurological outcomes, spinal extradural arachnoid cysts, spinal lesion

## Abstract

Spinal extradural arachnoid cysts (SEACs) are rare benign lesions that may cause progressive neurological deterioration due to cord compression. Surgical intervention is required for symptomatic cases, although the optimal surgical strategy remains unclear. We report the case of a 35-year-old female patient who presented with back pain, bilateral lower limb numbness, and progressive weakness leading to paraplegia. MRI revealed two thoracic extradural cystic lesions extending from the fifth to the ninth thoracic vertebrae (T5-T9) with cord compression. The patient underwent thoracic (T5-T9) laminectomy and complete excision of the cysts, along with closure of the intradural subarachnoid communication. Histopathology of the cyst walls confirmed arachnoid cysts. Postoperatively, the patient showed significant neurological recovery, achieving independent ambulation with only mild residual spasticity. A short video demonstrating complete excision of the arachnoid cysts and closure of the communication is shown in the article, providing a clear understanding of the surgical strategy for managing this rare disease. This case highlights the importance of early diagnosis and complete surgical excision with dural defect closure for optimal clinical outcomes in SEACs.

## Introduction

Spinal extradural arachnoid cysts (SEACs) are rare, benign lesions that account for approximately 1%-3% of all primary spinal space-occupying lesions [1,2]. They are most frequently located in the thoracic spine, particularly the middle or lower thoracic segments, and less commonly in the lumbar region, although they can occur at any level of the spine. SEACs arise from herniation or outpouching of the arachnoid membrane through a defect in the dura mater, forming a cerebrospinal fluid (CSF)-filled cavity within the extradural space [1,3].

The precise etiology of SEACs remains unclear. Both congenital and acquired mechanisms have been proposed, with potential contributing factors including trauma, inflammation, and prior spinal surgery [4,5]. SEACs most often occur in the thoracic region, usually in the posterior or posterolateral aspect of the spinal canal, and are more commonly observed in children and young adults [6,7].

Clinically, SEACs may remain asymptomatic for prolonged periods or present insidiously with symptoms due to mass effect on the spinal cord or nerve roots. Common manifestations include progressive compressive myelopathy [1,8]. Magnetic resonance imaging (MRI) is the diagnostic modality of choice, as it delineates the extent and location of the cyst while assessing the degree of spinal cord compression [3,7].

Symptomatic cyst needs treatment, but the optimal surgical strategy remains unclear and is decided by the surgeons’ preference. Cyst fenestration, marsupialization, or complete excision with dural repair has been described in the research literature. This approach generally results in favorable neurological recovery, especially if undertaken before irreversible cord injury occurs [2,5]. Delay in diagnosis or treatment, however, may result in permanent deficits.

Many previously reported studies have failed to demonstrate any intradural communication between the extradural arachnoid cyst and subarachnoid space. In this case report, we wish to present a case of multiple (two) spinal extradural arachnoid cysts with communication to subarachnoid space and their optimal surgical management.

## Case presentation

**Figure 1:**
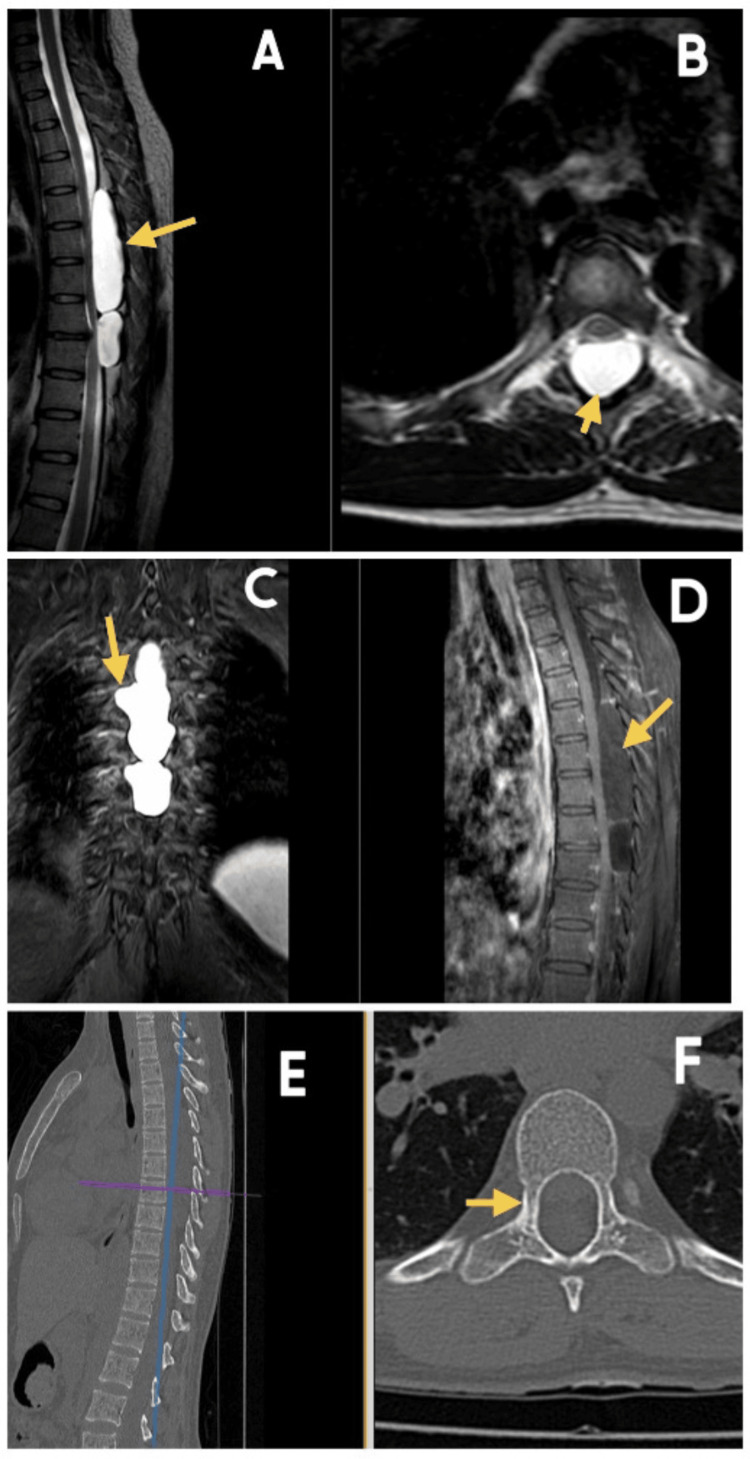
Preoperative imaging (A) T2-weighted sagittal MRI showing two well-defined large extradural hyperintense lesions causing compression over the underlying cord. (B) T2-weighed axial section. (C) Coronal T2-weighted MRI showing lateral extension of the arachnoid cyst into the foramen. (D) Contrast MRI sequence showing no postcontrast enhancement. (E) Sagittal CT scan of the dorsal spine. (F) Axial CT of the thoracic spine showing widening of the spinal canal and thinning of the pedicles.

We report the case of a thoracic SEAC extending, emphasizing the surgical management of this rare, benign lesion and its clinical outcome. A 35-year-old female patient presented with lower back pain and bilateral lower limb numbness for two months, associated with progressive difficulty in walking. The limb weakness worsened gradually, and by the time of admission, she was bedridden. There was no history of trauma. MRI of the thoracic spine revealed two well-defined posterior extradural cystic lesions extending from the fifth to the ninth thoracic vertebra (T5-T9), with lateral extension into the right T6-T8 neural foramina and anterior compression of the spinal cord. The findings were suggestive of SEACs (Type 1a spinal meningeal cysts). Computed tomography (CT) of the thoracic spine showed expansion of the spinal canal with thinning of the pedicles and lamina (Figure 1).

**Figure 2:**
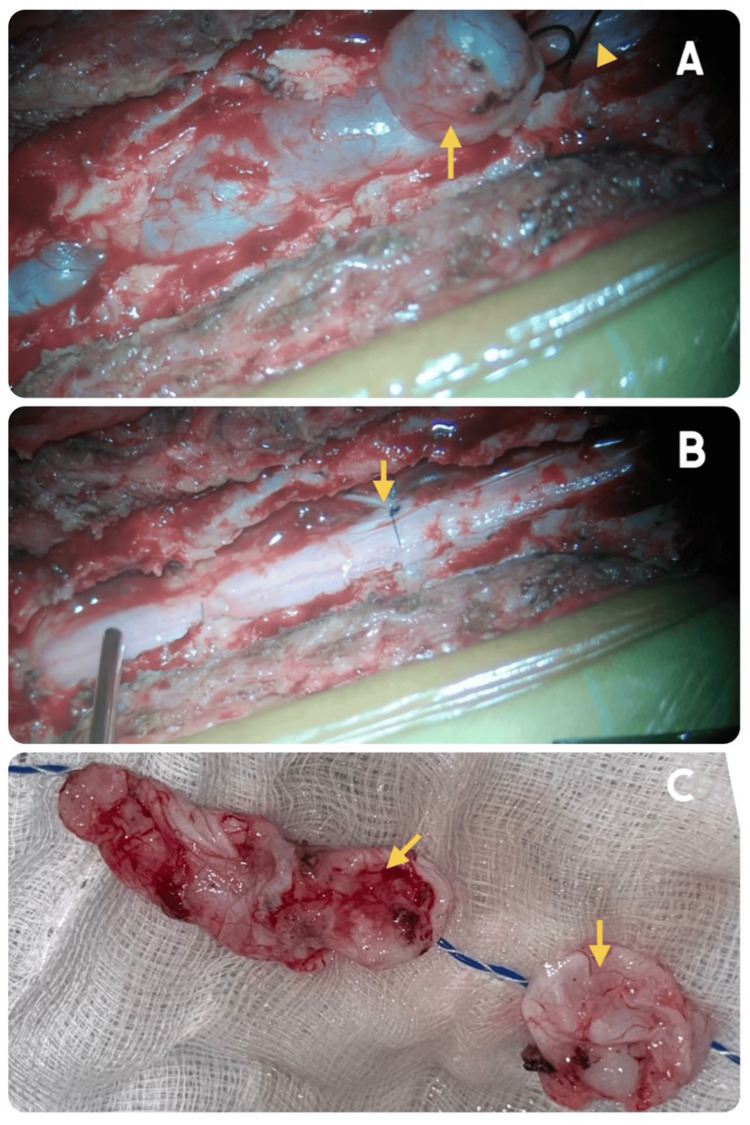
Intraoperative images (A) After laminectomy, the CSF-filled arachnoid cyst can be seen (yellow arrow) with the underlying dural sac (arrowhead). (B) Dural sac seen after complete excision. A Prolene suture (yellow arrow) was used to close the communication between the arachnoid cyst and dura. (C) Excised arachnoid cyst specimen.

The patient underwent thoracic (T5-T9) laminectomy with excision of the thoracic SEAC under intraoperative neuromonitoring. Intraoperatively, a large CSF-filled arachnoid cyst was identified and was easily separated from the underlying thecal sac. At the T6 and T8 levels, both cysts were found to communicate with the dural sac. These communications were cut and closed using 5-0 Prolene sutures. Following complete excision, the thecal sac appeared lax and pulsatile (Figure 2). Hemostasis was secured (Video 1).

**Video 1:** Two-dimensional intraoperative video showing complete excision of the spinal extradural arachnoid cyst

**Figure 3:**
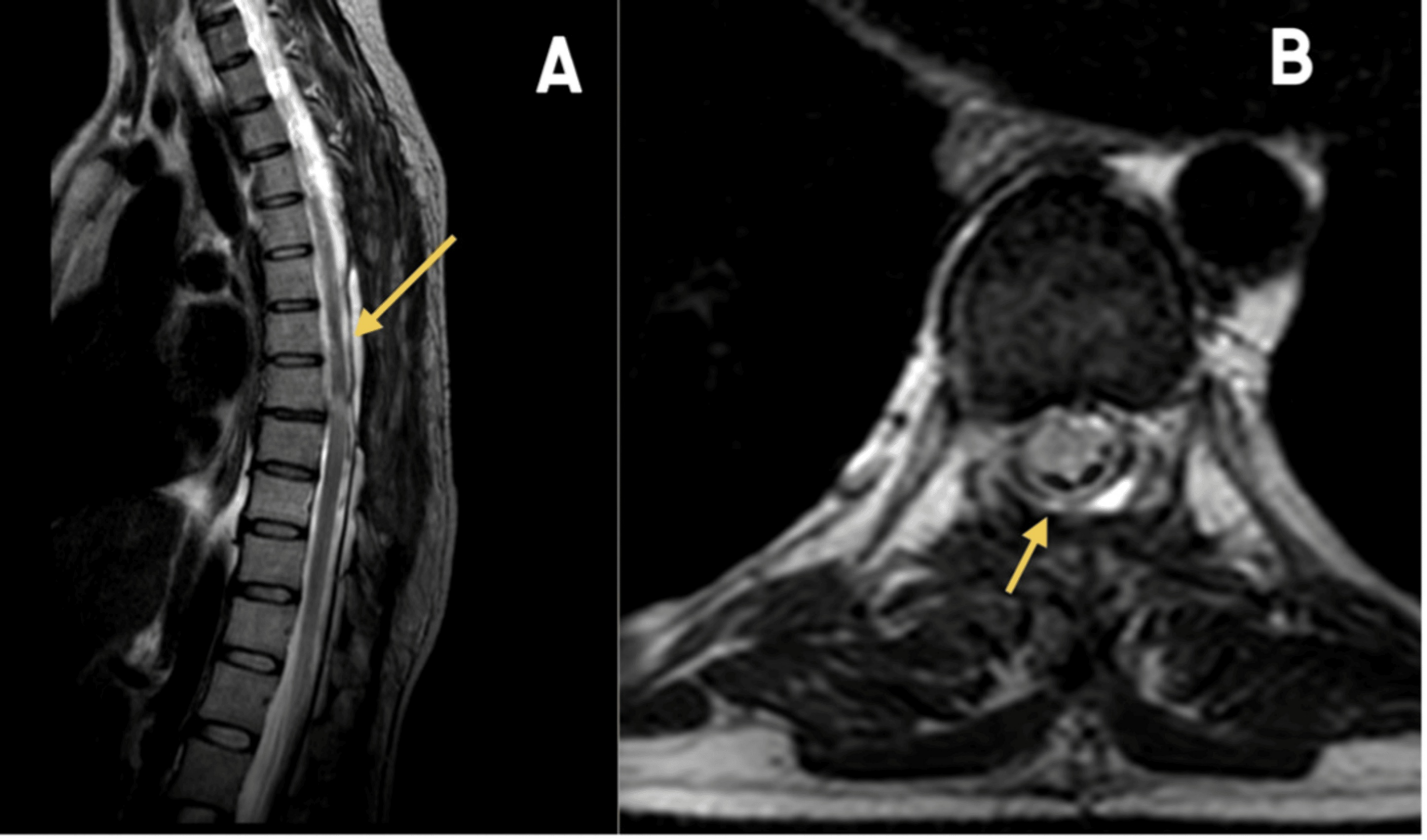
Postoperative images (A) Sagittal T2-weighted image showing complete excision. (B) Axial T2-weighted image.

The postoperative course was uneventful, and the patient was discharged on postoperative day 5. At follow-up, she demonstrated significant neurological improvement in lower limb strength and was able to stand and walk independently, though mild residual spasticity persisted in both legs (Video 2). Compared to the preoperative modified McCormick Grade of 6 (paraplegic), the patient improved to McCormick Grade 2 (independent) at follow-up. Postoperative MRI confirmed complete excision of the cyst (Figure 3).

**Video 2:** Video showing postoperative neurological recovery compared to the preoperative status

**Figure 4:**
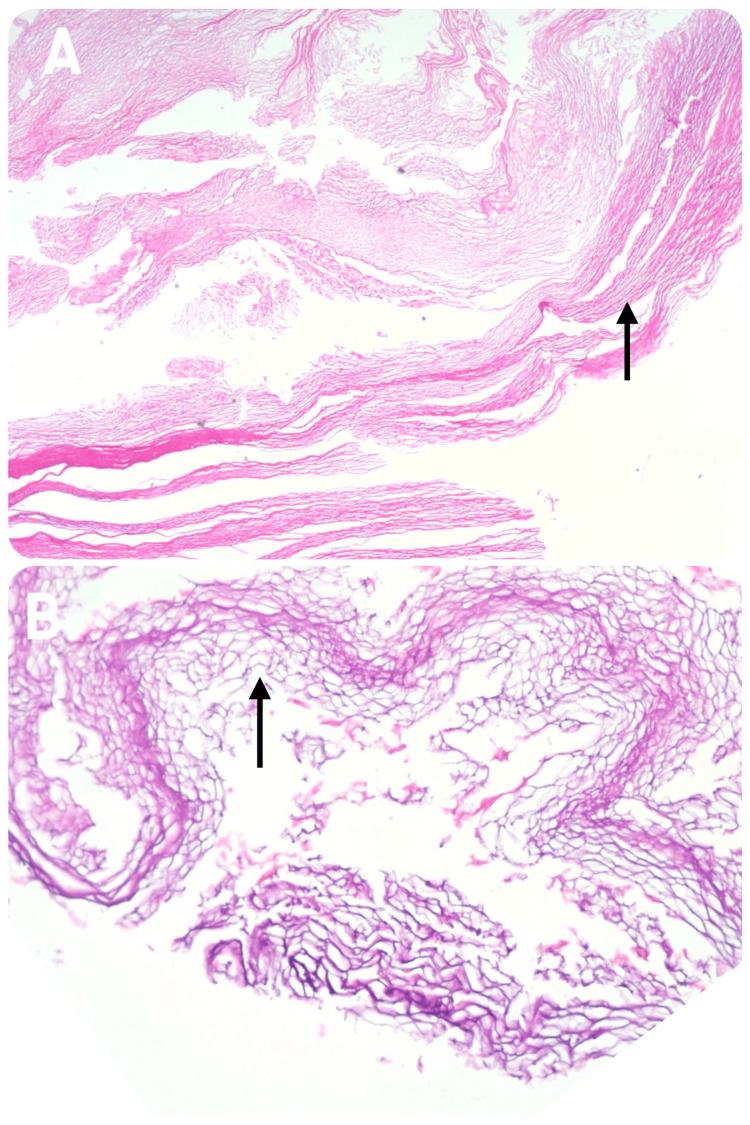
Histopathological images showing a thin fibrocollagenous cyst wall (A), focally lined by cuboidal meningothelial cells (B)

Histopathological examination of the cyst wall revealed thin fibrocollagenous tissue, focally lined by flattened meningothelial-like cells. These findings were consistent with an arachnoid cyst (Figure 4).

## Discussion

SEACs are rare lesions of the spine. They usually present as cystic formations caused by a dural defect and become enlarged through the flow of the CSF from the intradural arachnoid space [1,6]. Most SEACs are located posteriorly or posterolateral in the spinal canal, displacing the spinal cord anteriorly [1,6,8,9]. As they enlarge, SEACs may compress the spinal cord or nerve roots, leading to symptoms of compressive myelopathy such as pain, weakness, and sensory deficits [1,6,8,9].

Although multiple studies have investigated the etiology of extradural arachnoid cysts, their pathogenesis remains uncertain [1-9]. Most reports describe a communication between the subarachnoid space and the cyst [1,2,3,5,8]. This may be congenital or acquired. A small dural tear, caused by congenital anomaly, trauma, arachnoiditis, or prior surgery, allows CSF to herniate through the arachnoid membrane. These defects are commonly found near the nerve root sleeve [1,2,6]. Patients with structural abnormalities such as Marfan syndrome, dural ectasia, or with altered CSF dynamics due to trauma or leakage may be at greater risk [1,6,7].

The most widely accepted mechanism is that the dural defect acts as a ball valve, permitting CSF entry but not exit. Active secretion of CSF by residual arachnoid membrane may also contribute to cyst expansion [1]. This progressive enlargement leads to spinal cord compression. SEACs are classified into three types: Type 1, extradural arachnoid cysts without nerve root fibers (Type 1a: extradural arachnoid cysts; Type 1b: sacral meningoceles); Type 2, extradural arachnoid cysts with nerve root fibers; and Type 3, intradural meningeal cysts.

In our case, the patient had no prior history of trauma, arachnoiditis, or spinal surgery. Imaging and intraoperative findings revealed a dural defect at the T6 and T8 right root levels, with a trapped rootlet near the defect, consistent with the one-way valve mechanism described in earlier reports. The cyst corresponded to a Type 1 meningeal cyst. Histopathology demonstrated calcified tissue and layered collagenous fibers, without glandular or secretory tissue.

SEACs are often misdiagnosed because their symptoms overlap with those of more common degenerative spinal disorders. MRI is the most useful diagnostic tool, with SEACs typically showing low signal intensity on T1-weighted and high signal intensity on T2-weighted images, matching CSF [1,2,6,8]. CT myelography adds diagnostic value by more precisely defining the cyst location and the extent of neural compression [1]. Advanced CSF flow-sensitive MRI sequences may also help identify the site of dural communication, visualized as turbulent flow voids [1]. In this case, MRI accurately predicted the site of the defect, which was later confirmed surgically.

The optimal management of SEACs remains debated [1-9]. While some authors advocate conservative treatment with favorable outcomes [1,6], surgery is generally indicated when neurological symptoms develop or progress due to cyst enlargement [2,5]. There is agreement that closure of the dural defect is essential [1,2,3,6,9]. However, surgical approaches to the cyst vary. Some recommend complete excision to prevent recurrence [5], whereas others report success with cyst fenestration [1], aspiration, or shunting [5]. Multilevel laminectomy with total cyst excision and dural repair has long been considered the standard approach [1], but extensive laminectomy carries risks such as bleeding and postoperative spinal instability [1-9]. More recently, selective laminectomy with targeted closure of the dural defect has been reported as a safe and effective alternative [1,6].

In our case, we performed a thoracic laminectomy(T5-T9) with microsurgical excision of the cyst and dural repair. The dural connection of the cyst was clearly identified and closed after successful excision of the cyst. Postoperative imaging confirmed complete cyst resolution, and the patient experienced marked neurological recovery. As it is difficult to demonstrate the intradural connection of the arachnoid cyst on preoperative MRI, the video provided in this case report gives a clear understanding of the surgical strategy to separate the arachnoid cyst from the underlying dura and close the communication with the subarachnoid space. 

Table 1 summarizes previously published case reports on spinal extradural arachnoid cyst.

**Table 1:** Summary of published case reports and series and the present study on spinal extradural arachnoid cysts (SEACs) T: thoracic, L: lumbar, US: ultrasound.

No.	Author (Year)	Study type (no. of cases)	Spine level	Clinical presentation	Treatment	Outcome
1	Choi et al. (2013) [1]	Case report (2 cases)	Thoracolumbar (T12-L3)	Leg pain, paresthesia, compressive myelopathy	Laminectomy, cyst fenestration + dural repair	Symptom improvement; cyst resolution on imaging
2	Funao et al. (2012) [2]	Surgical series (several thoracolumbar cases)	Thoracolumbar	Back pain, radiculopathy, myelopathy	Resection ± dural repair	Good neurological outcomes
3	Lee et al. (2012) [5]	Case series (8 cases + literature review)	Thoracolumbar (variable)	Pain, motor deficits	Cyst removal vs fenestration; dural closure	Good outcomes; questioned need for total excision
4	Oh et al. (2012) [8]	Case series (14 cases)	Thoracolumbar	Pain, weakness, sensory loss	Laminectomy + closure of communication	Most patients improved
5	Furtado et al. (2011) [3]	Case series (4 "giant" cysts)	Thoracolumbar	Progressive myelopathy, paraparesis	Microsurgical excision ± dural repair	Symptomatic improvement
6	Netra et al. (2011) [6]	Case series (MRI evaluation)	Various	Pain, radiculopathy, myelopathy	Imaging study; surgical management discussed	MRI useful; surgery effective when symptomatic
7	Kanetaka et al. (2011) [4]	Case report	Thoracic	Back pain, motor weakness	Laminectomy + closure (guided by Doppler US)	Successful localization and good outcome
8	Ogura et al. (2013) [7]	Familial and sporadic cases (genetic study)	Thoracolumbar (multiple cysts)	Variable (including pediatric)	Surgery for symptomatic cases	Good surgical results; FOXC2 mutation reported
9	Panigrahi et al. (2012) [9]	Case report	Giant thoracolumbar cyst	Cord compression, paraparesis	Surgical excision + dural repair	Clinical and radiological improvement
10	Current study	Case report	Multiple thoracic (two- T5-T9)	Gradual paraparesis	Total resection + dural repair	Significant neurological improvement- patient independent

## Conclusions

SEACs are rare but clinically significant lesions. When identified and managed promptly, patients often achieve full recovery. Surgical strategy should focus on complete excision of the cyst wall, identifying and closing the intradural communication. In our patient, both clinical and radiological outcomes were excellent. At the one- and three-month follow-up, the patient showed complete recovery from compressive myelopathy and was able to mobilize independently with normal lower limb strength.
